# Migration risk of fall armyworm (*Spodoptera frugiperda*) from North Africa to Southern Europe

**DOI:** 10.3389/fpls.2023.1141470

**Published:** 2023-04-03

**Authors:** Jing Wang, Yanru Huang, Linsheng Huang, Yingying Dong, Wenjiang Huang, Huiqin Ma, Hansu Zhang, Xueyan Zhang, Xinyu Chen, Yunlei Xu

**Affiliations:** ^1^ National Engineering Research Center for Agro-Ecological Big Data Analysis and Application, Anhui University, Hefei, China; ^2^ Key Laboratory of Digital Earth Science, Aerospace Information Research Institute, Chinese Academy of Sciences, Beijing, China; ^3^ International Research Center of Big Data for Sustainable Development Goals, Beijing, China; ^4^ University of Chinese Academy of Sciences, Beijing, China; ^5^ School of Automation, Hangzhou Dianzi University, Hangzhou, China; ^6^ College of Plant Protection, Nanjing Agricultural University, Nanjing, China

**Keywords:** fall armyworm, invasion risk, migratory simulation, CLIMEX, HYSPLIT

## Abstract

With the development of globalization and agriculture trade, as well as its own strong migratory capacity, fall armyworm (FAW) (*Spodoptera frugiperda*) (J.E. Smith) has invaded more than 70 countries, posing a serious threat to the production of major crops in these areas. FAW has now also been detected in Egypt in North Africa, putting Europe, which is separated from it only by the Mediterranean Sea, at high risk of invasion. Therefore, this study integrated multiple factors of insect source, host plant, and environment to provide a risk analysis of the potential trajectories and time periods of migration of FAW into Europe in 2016~2022. First, the CLIMEX model was used to predict the annual and seasonal suitable distribution of FAW. The HYSPLIT numerical trajectory model was then used to simulate the possibility of the FAW invasion of Europe through wind-driven dispersal. The results showed that the risk of FAW invasion between years was highly consistent (*P*<0.001). Coastal areas were most suitable for the expansion of the FAW, and Spain and Italy had the highest risk of invasion, with 39.08% and 32.20% of effective landing points respectively. Dynamic migration prediction based on spatio-temporal data can enable early warning of FAW, which is important for joint multinational pest management and crop protection.

## 1 Introduction

The fall armyworm (FAW), *Spodoptera frugiperda* (J.E. Smith) is a notorious migratory pest native to the tropical and subtropical regions of the Americas (Suby et al., 2020). Due to the lack of diapause (Hardke et al., 2015), FAW can only successfully overwinter in areas with higher temperatures such as Florida and Texas, and wait until the weather warms before migrating north to obtain food (Johnson, 1987; Mitchell et al., 1991). In 2016, FAW was found in West African countries including Nigeria, Sao Tome and Principe, Togo, and Benin (Goergen et al., 2016; Sisay et al., 2018). With its strong migratory ability, it spread quickly to 44 African countries south of the Sahara Desert in the next two years, including Ethiopia, Tanzania, Uganda, Rwanda, Kenya, etc (Otim et al., 2018; Sisay et al., 2019; Elibariki et al., 2020; Matova et al., 2020). Its invasion and infestation have had a serious impact on local agriculture, with losses of $2531~6312 million in 12 major maize-planting countries in Africa (Day et al., 2017). Although EFSA Panel on Plant Health (EFSA PLH Panel) et al. (2018) believe that FAW cannot invade Southern Europe through a generation of migration from the sub-Saharan region. However, FAW may invade countries in southern Europe from North African countries such as Morocco and Tunisia through the wind as a medium (Shaw, 2016). FAW adults are extremely capable of migrating, they can migrate hundreds of kilometers in one night, and fly more than 500 kilometers in one generation (Westbrook and Sparks, 1986). In particular, the presence of FAW has been reported in maize fields in Egypt (Gamil, 2020), indicating an increased potential for invasion of Europe by FAW. Although FAW has been found in the Canary Islands which are part of Spain, Europe north of the Mediterranean has not yet become the breeding area for FAW. Therefore, there is an urgent need to indicate the periods and areas at high risk of FAW invasion in Europe, which will help in the early deployment of pest management measurements.

Although FAW has not yet invaded Europe, it is currently on the EPPO A1 list of pests recommended for regulation as quarantine pests by the European and Mediterranean plant protection organization due to its strong migratory, destructive, polyphagous, and reproductive abilities (EFSA Panel on Plant Health (EFSA PLH Panel) et al., 2018). Also, increasing studies have shown that there is a risk of FAW invasion in Europe. Early et al. (2018); Zacarias (2020); Ramasamy et al. (2022); Liu et al. (2020); Du Plessis et al. (2018), and Tepa-Yotto et al. (2021) explored the habitat suitability of FAW at different latitudes in Europe using species distribution models (SDM) such as MaxEnt, CLIMEX, etc. and indicated that the seasonal suitable distribution can extend from southern Europe to Ireland. Gilioli et al. (2022) assessed the risk of invasion by FAW in Europe based on a physiological demographic modelling approach and believed that the Mediterranean coastal areas of southern Europe were suitable for temporary FAW population establishment.

The long-distance migration of insects is often aided by favourable seasonal wind currents (Jia et al., 2022), such as the painted lady’s (*Vanessa cardui*) round-trip movements across the Sahara, and the brown planthopper’s (*Nilaparvata lugens*), beet armyworm’s (*Spodoptera exigua*), and oriental armyworm’s (*Mythimna separate*) south-north migration in China under the influence of the East Asian monsoon (Zhao et al., 2018; Hu et al., 2019). The use of numerical trajectory models is one of the most effective methods for simulating insect migration trajectories, such as The Hybrid Single-Particle Lagrangian Integrated Trajectory (HYSPLIT). This model was jointly developed by the National Oceanic and Atmospheric Administration’s Air Resources Laboratory (NOAA ARL) and the Australian Bureau of Meteorology, and has been used in recent years to forecast the wind-driven migration trajectories of many migratory insect species. Huestis et al. (2019) simulated long-distance migration of malaria mosquitoes in the Sahel using HYSPLIT. Westbrook et al. (2019) used HYSPLIT to simulate the multi-generational migration dynamics of FAW in the USA. In addition, insect migration is closely related to seasonal changes in environmental suitability and resource availability (Guo et al., 2020). Therefore, the use of remote sensing data and meteorological assimilation products to finely characterise the environmental conditions of insect habitats can help improve the accuracy of insect migration simulations (Huang et al., 2022).

On the basis of the above, this study combined multi-year spatio-temporal environmental data, and employed the species distribution model CLIMEX and the numerical trajectory model HYSPLIT to analyze the potential risk of FAW to invade European agricultural planting areas from North Africa across the Mediterranean Sea in 2016~2022. This study can help to plan FAW prevention and control measures in advance in southern Europe, to reduce the risk of FAW outbreaks and ensure food security.

## 2 Materials and methods

### 2.1 Study area and FAW occurrence data

#### 2.1.1 Study area

**Figure 1:**
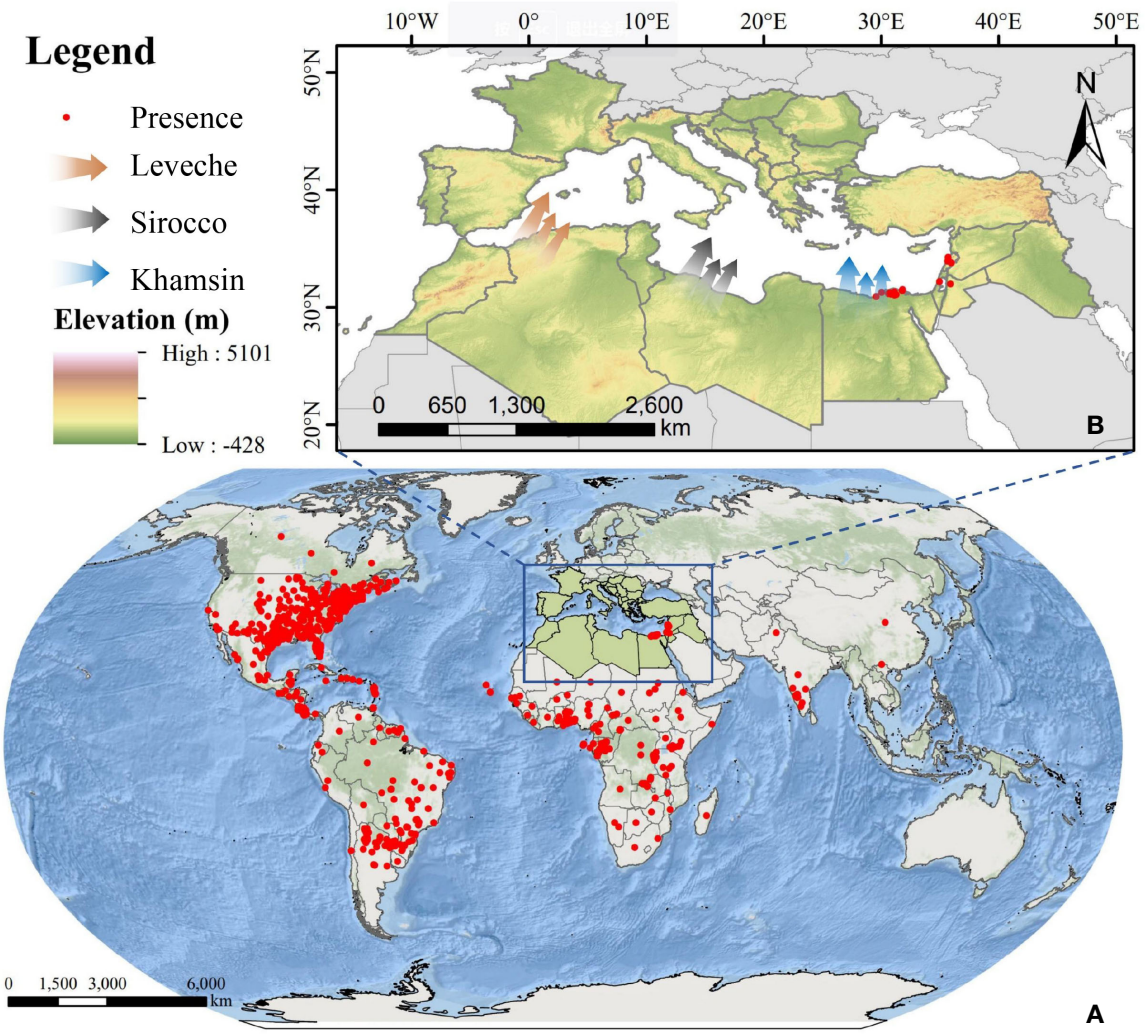
Overview of the study area; **(A)** global occurrence data of FAW; **(B)** overview of the study area in North Africa and Southern Europe and three types of winds favorable for FAW migration to Europe (red: Leveche, grey: Sirocco, blue: Khamsin).

The study area is located in North Africa and Southern Europe, as shown in **Figure 1**. The Mediterranean coast, the Nile River coast, and the estuary deltas in North Africa have developed irrigated agriculture, which can provide suitable environment for FAW to survive. The topography of the area is homogeneous and flat, which is topographically advantageous for the outward migration of FAW (Houssaine et al., 2020). Also, the low-level jet winds blowing from the south (Forrester, 1982) can provide a suitable medium for the migratory activities of FAW, such as Leveche, Sirocco, and khamsin(Stefanescu et al., 2007; EFSA Panel on Plant Health (EFSA PLH Panel) et al., 2018). The southern European countries of Spain, Italy, Greece, and Turkey are separated from North Africa by the Mediterranean Sea. These areas have developed a unique Mediterranean agriculture, where the main crops planted are wheat, maize, and rice, which are the preferred host crops of FAW, and the sufficient food supply provides an effective guarantee for the life activities of FAW after its invasion (Migliorini et al., 2018). Based on the above environmental conditions, it is possible for FAW to migrate long distances across the sea from North Africa to Europe.

#### 2.1.2 FAW occurrence data and data cleaning

This study obtained the historical occurrence records of FAW from the Global Biodiversity Information Facility (GBIF, www.gbif.org), with a total of 3800 geographical location records from 1877 to 2022. In addition, we also obtained the location of the occurrence of FAW in some African countries such as Ethiopia, Kenya, Tanzania, and Nigeria from the published articles (Goergen et al., 2016; Nagoshi et al., 2017; Early et al., 2018; Nagoshi et al., 2018). For the collected data, we carried out data pre-processing to remove missing and duplicate data. To avoid spatial autocorrelation, we used SDMtoolbox2.0 (www.sdmtoolbox.org) to keep only one occurrence record in every 10′ grid cell (Brown et al., 2017; Huang et al., 2020), and finally obtained a total of 1165 occurrence records for predicting the annual and seasonal suitable distribution of FAW.

### 2.2 Climate data and remote sensing data

#### 2.2.1 Climate data

The climate data for predicting the suitable distribution of FAW were obtained from the CliMond historical climate dataset (Kriticos et al., 2012) (www.climond.org) with a spatial resolution of 10′, which was generated by reformatting, adjusting, and combining the underlying historical data from Worldclim and the Climate Research Unit (CRU), including daily minimum temperature (Tmin), daily maximum temperature (Tmax), monthly precipitation (Rainfall), and relative humidity at 9:00 (RH 0900) and 15:00 (RH 1500).

#### 2.2.2 Atmospheric condition data

Atmospheric condition data were obtained from the Global Data Assimilation System (GDAS) (ftp.arl.noaa.gov), which puts surface observations, balloon data, wind profiler data, aircraft reports, buoy observations, radar observations, and satellite observations into a gridded, 3-D, model space to generate global atmospheric condition data, with a spatial resolution of 1°× 1° and a temporal resolution of 3 hours, including temperature, precipitation, and wind speed in vertical and horizontal directions, which can provide near-real-time meteorological data for the simulation of the migration trajectory of FAW.

#### 2.2.3 Vegetation phenology data

Vegetation phenology data (GLSP) were obtained from the Land Process Distributed Active Archive Center (LPDAAC) (https://lpdaac.usgs.gov/) with a spatial resolution of 500m. It generates two-band Enhanced Vegetation Index (EVI2) time series based on the daily VIIRS Nadir Bidirectional Reflectance Distribution Function (BRDF)-Adjusted Reflectance (NBAR) to characterize the vegetation growth. This product identifies six key phenological transition dates (Zhang, 2015; Zhang et al., 2018) by vegetation index curvature changes, which are the onset of greenness increase, onset of greenness maximum, onset of greenness decrease, onset of greenness minimum, dates of mid-greenup, and senescence phases. From these, we extracted the times and locations of the onset of greenness increase (emergence) and senescence phases (dormancy) in the study area to determine the spatial and temporal range available for FAW to obtain food resources and carry out its life history.

### 2.3 FAW Spatio-temporal migration simulation method

This study combined spatio-temporal big data and integrated insect sources, host plants, and environment to explore the risk of invasion of Europe by FAW migrating from North Africa, which consisted of three main parts. Firstly, the species distribution model CLIMEX was used to predict the suitable distribution of FAW, based on its climatic preferences and biological parameters; Secondly, the starting points of forward migration were determined through the suitable seasonal distribution of FAW in North Africa, and based on the numerical trajectory model, a series of possible migration trajectories were simulated by combining the migration characteristics of FAW and atmospheric conditions; Finally, we monitored the vegetation conditions at the landing points of the migration trajectories based on remote sensing products, and the effective landing sites had the vegetation in the growing season and the vegetation could meet the developmental needs of FAW for at least one generation.

#### 2.3.1 The annual and seasonal suitable distribution prediction of FAW

In this study, the CLIMEX model (Version 4, Sutherst, 1985; Kriticos et al., 2015) was used to predict the annual and seasonal suitable distribution of FAW to identify potential take-off and invasion sites. Environmental factors have both positive and negative effects on FAW survival, and the CLIMEX model divides climate parameters into Growth index (GI) and Stress index (SI). GI includes the optimum range of temperature and soil moisture for FAW growth, development, and reproduction, and also takes into account the biological characteristics and effective cumulative temperature. SI reflects the performance of FAW under unsuitable survival environment, and there were four main stress indicators involved in this study, namely Cold Stress (CS), Heat Stress (HS), Dry Stress (DS), and Wet Stress (WS). GI and SI were combined to determine the Ecoclimatic index (EI), which we used to judge the annual suitability of FAW. The EI ranges from 0 to 100 and is classified into four categories with reference to Paudel Timilsena et al. (2022): EI=0 means the area is not suitable for the long-term survival of FAW; 0<EI ≤ 10 means low suitability and limited conditions for FAW long-term survival; 10<EI ≤ 30 means moderate suitability and the area can accommodate a large number of population of FAW; EI>30 means high suitability and the area has very favorable conditions for the survival of FAW. The GI was used to evaluate the seasonal suitability for areas with EI of 0. These areas do not have suitable climatic conditions for overwintering of FAW, but some periods of the year may be suitable for FAW to survive, resulting in a seasonal infestation. Therefore, for regions with EI=0 and GI>0, we also divided them into three categories, with EI=0, 0<GI ≤ 10 indicating low suitability, EI=0, 10<GI ≤ 30 indicating moderate suitability, and EI=0, 30<GI ≤ 100 indicating high suitability.

In this study, we used the location comparison function of the CLIMEX model to estimate the climatic conditions required for the survival of FAW. Wood et al. (1979) found that the development of FAW began at temperatures higher than 12°C, so we set the lower temperature threshold to 12°C. Busato et al. (2005) found that FAW reared in a constant temperature box at 25°C in the laboratory rarely showed abnormalities, and the reproductive capacity and lifespan of the adults were the strongest compared to those reared under other conditions. Therefore, we set the lower optimal temperature to 25°C. Du Plessis et al. (2020) found that the development rate of FAW increases with temperature from 18°C until it reaches 30°C, after which the development rate decreases. Therefore, we set the upper optimal temperature to 30°C. Simmons (1993) reported that more than half of FAW reared in a constant temperature box at temperatures higher than 35°C showed body deformities and died within the next 24 hours. Therefore, we set the upper temperature threshold to 36°C. We referred to the research data of Du Plessis et al. (2018) regarding the relationship between FAW growth and development and soil moisture. Accordingly, we set the lower soil moisture threshold to 0.15, the lower optimal soil moisture to 0.8, the upper optimal soil moisture to 1.5, and the upper soil moisture threshold to 2.5. The effective accumulated temperature required for the growth and development was determined to be 391.61°C based on the research data of Du Plessis et al. (2020) on FAW. We iteratively adjusted the stress parameters in the model based on the known annual suitable distribution and seasonal suitable distribution of FAW, to obtain a predictive model of the FAW’s distribution that best fits the actual distribution. The final parameters are shown in **Table 1**. The output of the model is the EI and GI values corresponding to 565,801 stations worldwide. By setting the search radius as 0.25° through the Inverse Distance Weighted method (IDW), the discrete results predicted by the CLIMEX model are spatially interpolated to obtain the annual and seasonal suitable distribution of FAW with a spatial resolution of 0.05°.

**Table 1:** CLIMEX parameter values used for FAW suitable distribution modeling.

Indicators	Parameter	Description	Value	Unit
Moisture	SM0	Lower soil moisture threshold	0.15	/
	SM1	Lower optimal soil moisture	0.8	/
	SM2	Upper optimal soil moisture	1.5	/
	SM3	Upper soil moisture threshold	2.5	/
Temperature	DV0	Lower temperature threshold	12	°C
	DV1	Lower optimal temperature	25	°C
	DV2	Upper optimal temperature	30	°C
	DV3	Upper temperature threshold	36	°C
Cold Stress	TTCS	Cold stress temperature threshold	8	°C
	THCS	Cold stress accumulation rate	-0.005	week^-1^
Heat Stress	TTHS	Heat stress temperature threshold	39	°C
	THHS	Heat stress accumulation rate	0.0025	week^-1^
Dry Stress	SMDS	Soil moisture dry stress threshold	0.1	/
	HDS	Dry stress accumulation rate	-0.005	week^-1^
Wet Stress	SMWS	Soil moisture wet stress threshold	2.5	/
	HWS	Wet stress accumulation rate	0.01	week^-1^
Degree Days	PDD	Degree days per generation	391.61	°C

#### 2.3.2 The dynamic migratory trajectory simulation of FAW

Based on the predicted suitable distribution of FAW obtained from the CLIMEX model, this study divided FAW seasonal suitable distribution into several spatial units with a resolution size of 1°×1° to ensure that the starting points of the forward migration trajectory were evenly distributed in each spatial unit. Then, the HYSPLIT numerical trajectory model (Version5.2.0, https://ready.arl.noaa.gov/HYSPLIT.php) was used to analyze the main migration trajectories and time of FAW. We added the insect’s self-flight speed to the model by modifying the SETUP file, and implemented batch simulation of insect trajectories based on a localized trajectory calculation module using Python 2.7. April to August is the agricultural planting season in North Africa (https://ipad.fas.usda.gov/ogamaps/cropcalendar.aspx), so this study conducted a daily migration simulation of FAW during this period.

According to the biological characteristics of FAW, we set the autonomous flight parameters for the migration simulation. FAW are nocturnal moths with multi-stop migration habits (Li et al., 2020), usually taking off at dusk, migrating long distances at night, and terminating their flight at dawn the next day (Chapman et al., 2010; Wang et al., 2017). Since the study area spans multiple time zones, this study calculated sunset time for each migration starting points based on date and geographic location, and set it as the take-off time. The flight duration is 10 hours. If the weather conditions are favorable, FAW can fly for three consecutive nights (Wu et al., 2022). Also, we considered unfavorable weather conditions to terminate insect migration activities (Wu et al., 2018). By observing the migration behavior of FAW, it will stop flying when the air temperature is below 13.1°C (Chen et al., 2022). When the rainfall was greater than 1 mm/h, the moths will also be forced to land (Wu et al., 2021). In addition, because the migration from North Africa to Southern Europe crosses the Mediterranean Sea, when the flight stops with the landing sites at sea, FAW will be forced to extend the duration of a single flight, and the maximum duration of a single flight was set to 36 hours (Ma et al., 2019), Chen et al. (2022) also indicated that the maximum single flight time of FAW could reach 36.5 hours. Westbrook (2008) monitored the migration process of FAW by radar and noted that it generally migrates at altitudes with wind speeds greater than 10 m/s. Therefore, we combined the characteristics of the flat terrain in North Africa and set up flight altitudes every 250m from 500m~2250m, for a total of 8 groups. The vertical movement of FAW during migration was chosen to be isobaric, i.e., the migration was always maintained at the same air pressure conditions as the take-off altitude, and the maximum altitude was set to 3000m (Westbrook et al., 2016). Noctuidae insects of similar size to FAW fly at about 2.5~4.0 m/s, so the autonomous flight speed of FAW was set at 3 m/s in the same direction as the wind (Minter et al., 2018).

**Table 2** lists the autonomous flight parameters used for the migration simulation of the HYSPLIT model in this study. We combined the GDAS atmospheric data to simulate the migration process of FAW from April to August 2016-2022. The vegetation growth period from greenness increase to senescence phases in the invasion area was further determined from the phenological data to screen for effective landing points where the vegetation condition was appropriate. Specifically, the GLSP contains the Day of Year (DOY) for different phenological stages of vegetation. We selected the emergence and dormancy periods as the two key phenological stages and recorded the DOY when the host plants reached these stages. Then, for the effective landing points where the FAW arrived in the host plant planting areas, we compared the landing date with the DOYs, and only retained the landing points where the arrival time occurred after the emergence period and was more than 30 days before the dormancy period, to ensure that the host plants in the invaded area were sufficient to support the survival of one or multiple generations of FAW. By comparing the results of interannual migration, we obtained the possible distribution range and time periods of the invasion of FAW into Europe. Then, using the kernel density analysis function in ArcGIS 10.6, based on the density distribution of effective landing points, we mapped the annual invasion risk distribution of FAW, which can also reflect the relative abundance of FAW populations.

**Table 2:** Parameters used for simulating FAW migration.

Parameter	Value	Reference
Height	500 750 1000 1250 1500 1750 2000 2250m	Westbrook, 2008
Single run time	10h	Li et al., 2020
Direction	Forward	/
Top of model	3000 m AGL	Westbrook et al., 2016
Vertical motion method	isobaric	Westbrook et al., 2016
Duration	3 days	Wu et al., 2022
Self-velocity	3m/s	Minter et al., 2018
Temperature threshold	13.1°C	Chen et al., 2022
Max rainfall threshold	1 mm/h	Wu et al., 2021
Max flight time	36h	Ma et al., 2019

### 2.4 Atmospheric migration pattern analysis of FAW

To quantify the extent of the area affected by the migratory activity of FAW, we used a 2-level standard deviation ellipse algorithm in ArcGIS 10.6 for the effective trajectories landing points. The ellipse was based on 95% of the effective landing points, and its center, the length of the long and short axes, and the angle of rotation relative to due north could indicate the location, range, and directional distribution of the potentially affected area. Specifically, the elliptical axis indicates the range of the affected distribution, and the ratio of the long axis to the short axis can indicate the dispersal direction and trend of FAW, the larger the ratio, the more obvious the trend of the landing points distribution, if the short axis and the long axis are equal, the landing points distribution is directionless. We used paired t-test to compare whether long and short axes of the standard deviation ellipse are significantly different by month and year respectively, to determine whether the distributions are significantly directional.

In addition, we studied the correlation of migratory flight activities of FAW at different time scales. First, for the effective landing points obtained from the simulation, we plotted interannual and inter-monthly box line plots respectively, which can clearly represent the distribution of landing points in different periods. To express the migration of FAW between different years, we compared the correlation and significance between the interannual distributions by raster correlation analysis. The analysis is implemented in ‘corrplot’ in R version 4.1.0.

## 3 Results

### 3.1 The annual-seasonal suitable distribution of FAW

**Figure 2:**
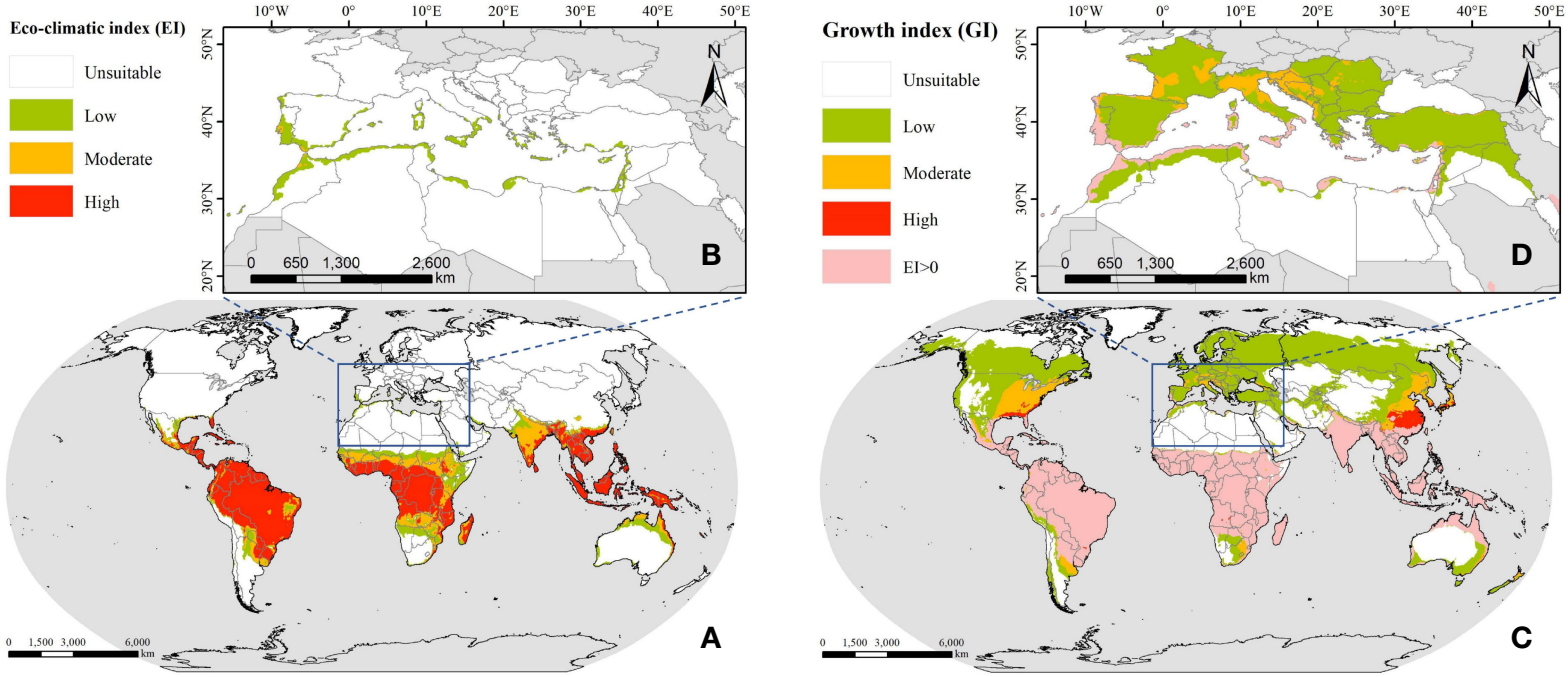
The suitable distribution of FAW predicted by CLIMEX; **(A)** annual suitable distribution in the world; **(B)** annual suitable distribution in the ROI; **(C)** seasonal suitable distribution in the world; **(D)** seasonal suitable distribution in the ROI.

To obtain the facially continuous suitable distribution of FAW, we performed IDW interpolation on the results predicted by the CLIMEX model, with the search radius set to 0.25° and the output results with a spatial resolution of 0.05°, as shown in **Figure 2**. The predicted annual suitable distribution results show that South America, sub-Saharan Africa, and most of Southeast Asia are suitable for the annual breeding of FAW, and these areas have tropical and subtropical climates, which can provide sufficient heat for the growth and breeding of FAW even in the coldest months of the year. When considering the seasonal suitable distribution, it can be seen that the climatically suitable range expands northward, with the temperature being the most influential factor limiting their year-round reproduction to one or more generations. Thus, FAW expands to higher latitudes in summer when temperatures rise, and waits until autumn when temperatures drop before migrating south.

For North Africa, the high temperatures and drought stress in the Sahara desert greatly restricted the establishment of permanent breeding populations of FAW. Only the northern part adjacent to the Mediterranean Sea, which had a Mediterranean climate and strong local irrigated agriculture, met the conditions for the survival and development of FAW. The nine countries along the Mediterranean coast had a total area of 277.78 thousand km^2^ suitable for the annual breeding of FAW, accounting for about 4.46% of the national territory, of which 267.95 thousand km^2^ were of low suitability and the rest were of medium suitability. The seasonal suitable distribution increased 149.87% compared with the annual suitable distribution, with Morocco, Algeria, and Libya having the largest suitable distribution areas of 274.63 thousand km^2^, 202.11 thousand km^2^, and 86.34 thousand km^2^, respectively. For southern European countries, despite being located at mid to high latitudes where low-temperature stress prevented the establishment of permanent populations of FAW, it was possible that in some Mediterranean climate regions, 1~3 generations of FAW may be available for some months of the year (EFSA Panel on Plant Health (EFSA PLH Panel) et al., 2018).

### 3.2 Spatio-temporal risks of FAW migration to Europe

We divided the seasonally suitable distribution area of FAW in North Africa based on the CLIMEX model into several 1° × 1° grid cells, and identified 67 migration starting points (**Figure S1**) from which we simulated a total of 686,616 migration trajectories from 2016~2022, with an average of 98,088 trajectories per year, and finally screened 33,663 effective landing points.

Depending on the potential routes of FAW invasion in southern Europe, we divided the starting points into 3 parts. The first part was located in Morocco, Algeria, and Tunisia. Some of the FAW would cross the Strait of Gibraltar into Spain and Portugal, and then spread from Spain to France; others would cross the Ligurian Sea and Tyrrhenian Sea to Italy, and invade the interior of France *via* Majorca, Sardinia, and Corsica. The majority of potential invasion sites were located in Spain and Italy, with 48.91% and 34.62% of effective landing points, respectively, followed by France (8.41%) and Portugal (6.37%). The second part was located in Libya, with a wide range of potential landing sites. Some FAW would cross the Ionian Sea to Italy and invade Montenegro and Albania *via* the Adriatic Sea, others would enter Greece *via* the Mediterranean Sea and then spread to western Turkey. A few FAW could reach as far as Romania after three consecutive nights of flight. Italy, Greece, and Turkey were at greater risk of invasion by FAW with 39.69%, 38.03%, and 12.49%, of effective landing points, respectively. The third part was located in Syria and Lebanon on the border between Egypt and Asia and Africa, FAW would first reach Cyprus and then enter Turkey, and some would directly invade Turkey. 83.05% of the effective landing points were located in Turkey, mainly in the eastern region, with a few effective landing points in Cyprus (9.20%) and Greece (6.65%).

The migration of FAW varied by month due to wind and vegetation phenology. In April, effective landing points were mostly concentrated in central and eastern southern Europe, in June and July effective landing points were mostly concentrated in central and western parts, while in May and August have a wider longitude distribution, spanning about 80 longitude bands. The risk of invasion by FAW also varied by month in different countries. The risk of invasion by FAW was more frequent in Spain, Portugal, and France from May to August, and rarely in April; Greece was more likely to be invaded by FAW from April to July, and the risk decreases after August; while Italy and Turkey were at risk of invasion by FAW from April to August.

**Figure 3:**
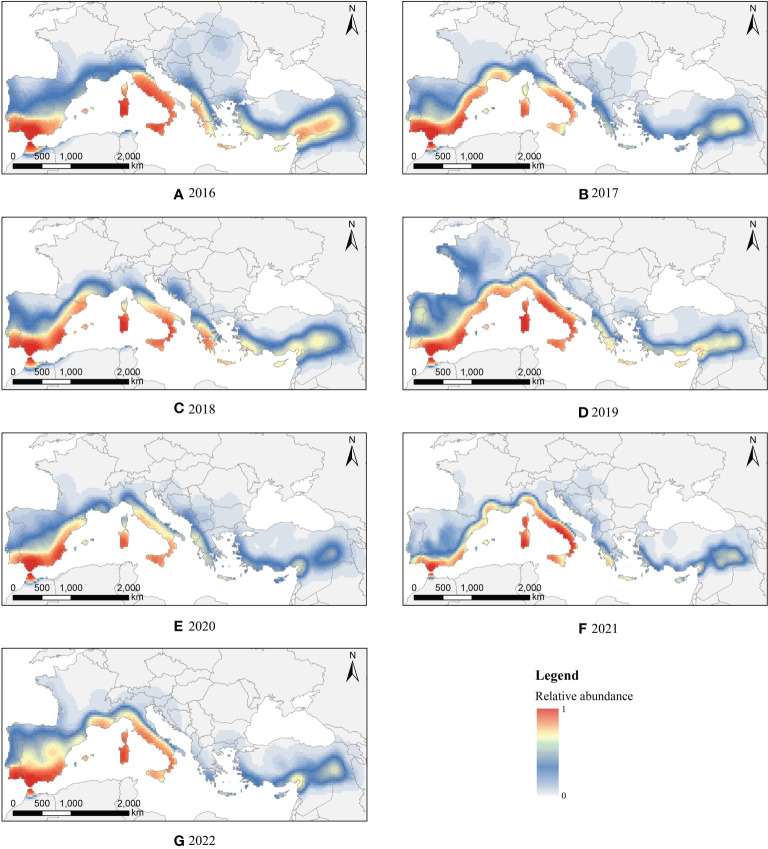
Invasion risk distribution of FAW in southern Europe from **(A–G)**: 2016 to 2022.

We conducted kernel density analysis on the effective landing points, to obtain the risk distribution map of FAW invasion in southern Europe from 2016 to 2022, as shown in **Figure 3**. The multi-year simulation result showed that the risk of invasion was significantly higher in coastal areas than inland. By quantitatively counting the ratio of effective landing points in each country to the total effective landing points, the risk of each country being invaded is compared. Spain had the highest risk of invasion; it was separated from Morocco only by the Strait of Gibraltar, and FAW can enter Spanish territory by a single night flight. The top six countries from highest to lowest risk of invasion were Spain(39.08%), Italy(32.20%), Turkey(8.89%), France(6.82%), Greece(5.77%), and Portugal(5.08%), and their combined risk of invasion reached 97.84%.

### 3.3 Migration patterns of FAW in different time periods

**Figure 4:**
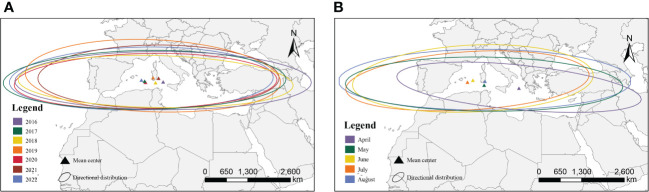
Simulated affected areas for the migration of FAW; **(A)** interannual affected areas; **(B)** monthly affected areas.

We compared the sites affected by FAW in different years and months according to the 2-level standard deviation ellipse method (**Figure 4**). The annual map showed that the distribution of the centroids was concentrated with little variation in the latitudinal range of the affected areas. However, the monthly map showed that the distribution of the centroids was discrete, and the affected area varies widely between months. The directional angle of the ellipse ranged from 85.06° to 98.92°, and the mean ratio of the long and short axes was 6.60, with significant directionality (paired t-test for long and short axes, t=22.98, n=34, P<0.001).

**Figure 5:**
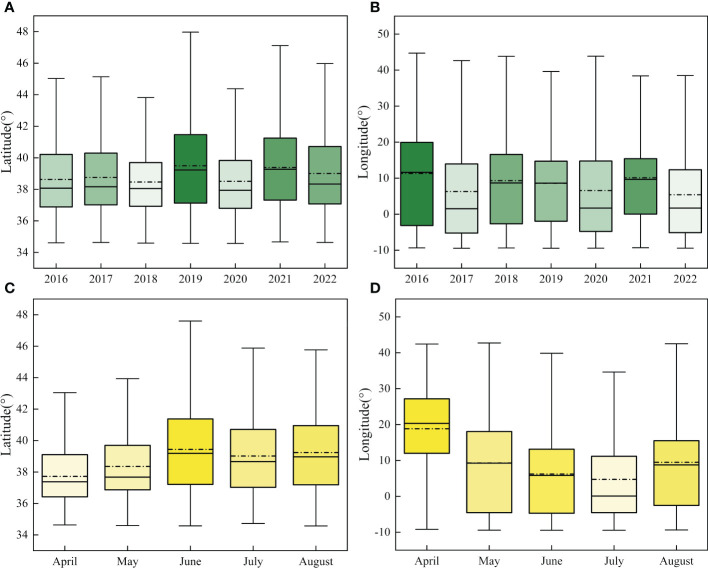
The boxplot of effective landing points distribution; **(A)** Latitude distribution in different years; **(B)** Longitude distribution in different years; **(C)** Latitude distribution in different months; **(D)** Longitude distribution in different months.

To quantify the geographic distribution of FAW invading Europe, we created annual and monthly box line maps for the latitude and longitude information of the effective landing points, as shown in **Figure 5**. The monthly distribution of effective landing points varied significantly, with the invasion range of FAW gradually moving northward over time, peaking in June and decreasing slightly later, mostly concentrated at 36°N~42°N. Among them, the effective landing points were mostly concentrated in 3°W~20°E, and the latitudes were slightly higher in 2019 and 2021 than in other years, with FAW migrating to 48°N in 2019. Also, in April, the migration of FAW was concentrated in the eastern part of southern Europe, then gradually moved westward and concentrated in the central part of the country in August.

In general, the migration behavior of FAW was mostly different on a monthly scale, and the interannual trend was relatively consistent. We conducted a raster correlation test on the results of the kernel density analysis between years for FAW, and the results showed that the invasion risk distributions were significantly correlated between years (P<0.001). Therefore, we believe that it is not a coincidence that FAW is at risk of invading Europe, and there are suitable environmental conditions every year for FAW to invade southern European countries by wind-driven migration, as well as suitable climatic and vegetation conditions for its survival and reproduction.

## 4 Discussion

In this study, we analyzed the risk of FAW invasion to southern Europe from 2016 to 2022 by wind-driven migration from North Africa, as well as invasion trajectories and time periods. Firstly, we predicted the potential suitable distribution of FAW, and the results were generally consistent with Du Plessis et al. (2018), but there were some differences. Most non-desert areas in tropical and subtropical climate zones worldwide are suitable for the year-round survival of FAW, such as most areas in South America, Africa south of the Sahara Desert, and Southeast Asia. The differences mainly lie in the boundary between annual suitable distribution and seasonal suitable distribution. Our annual suitable distribution extends further south in South America and Africa and further north in India. This is consistent with the research results of Senay et al. (2022), who pointed out that countries such as Angola, Botswana, Namibia, and some areas along the eastern coast of South Africa, as well as high-latitude areas in India, can support the year-round survival of FAW. In addition, other studies have used the MaxEnt model to predict the global potential distribution of FAW. Ramasamy et al. (2022) simulated the potential distribution of FAW based on climate indicators set, and the distribution results in North America, South America, and Africa were generally consistent with those predicted by the CLIMEX model. However, the mean annual temperature limits the suitability of high latitude regions in the Northern Hemisphere. Similarly, Liu et al. (2020) used the MaxEnt model and considered land use types, concluding that the impact of land use changes on the suitable distribution of FAW is significant. Therefore, based on our current research, the next step in improving the model would be to incorporate factors such as land use types and soil conditions.

Although FAW could not establish an annual breeding area in most of North Africa due to the high temperature and drought conditions of the Sahara Desert. However, the Mediterranean coast and some areas of the Nile basin can support year-round reproduction of FAW due to the development of irrigated agriculture, coupled with suitable climatic conditions and abundant planting crops that provide suitable habitat for the survival of FAW. At present, FAW has been found in the fields of Aswan Governorate in southern Egypt, and it is likely that FAW will expand its invasion area westward through dispersal in the near future, and gradually establish populations in these areas. At this time, the risk of invasion by FAW will increase in southern European countries, and our species distribution model predictions also suggest that the climatic conditions in southern Europe are suitable for the seasonal survival of FAW.

FAW has a strong migration ability. According to the sea-trapping experiments of Baust et al. (1981) and Sparks et al. (1986), it was demonstrated that FAW could cross the Gulf of Mexico by migration. Ma et al. (2019) predicted the routes of FAW invasion across the sea from China to Japan and Korea by trajectory analysis model and verified their results by field survey data. Our results showed that under suitable monsoon conditions, FAW can successfully invade southern Europe from North Africa through one generation of migration. Spain and the Italian island of Sardinia are the closest to North Africa, and especially Spain and Morocco are separated by only the Strait of Gibraltar, the shortest distance of which is only 14 km, and are therefore at the highest risk of invasion by FAW. In addition, most of FAW need to migrate across the Mediterranean Sea to invade southern Europe, so coastal areas are at higher risk of invasion than inland areas.

The migratory behavior of insects is mainly an ecological adaptation strategy in time and space to escape from unfavorable environmental conditions and natural enemies, and to seek abundant host crops to sustain their survival. The time period of invasion was also geographically varied by vegetation phenology. The risk of invasion of Italy, Greece, and Turkey was high in April, and in May the western countries such as Spain and Portugal also started to suffer from the risk of invasion. By August, the risk of invasion in Greece was gradually reduced, while Spain was still at high risk. The results of the annual invasion risk distribution showed a relatively consistent trend between years, suggesting that the simulation results are not a coincidence and that southern Europe would be at risk of annual invasion by FAW if it colonized North Africa. If pest management measures are not taken in time, it may cause great damage to local crops, especially maize, as the majority of FAW in Africa have been identified as the maize strain (Tendeng et al., 2019).

**Figure 6:**
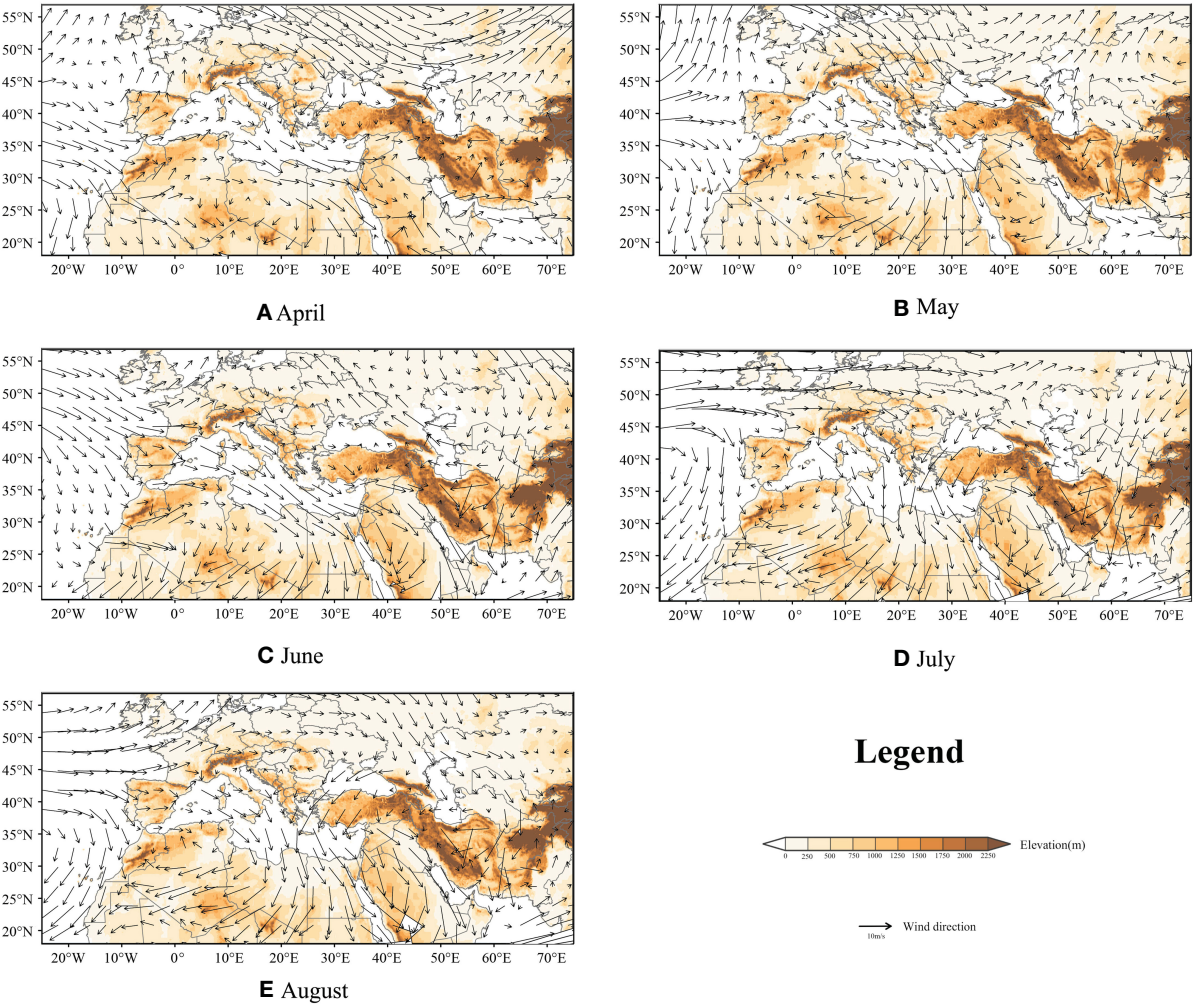
850hPa mean night atmospheric conditions from April to August; background represents topography; and arrows represent wind direction and speed; **(A–E)**: April to August.

For FAW, wind is an important factor in their migratory activity, as self-powered flight can only support their short-range dispersal. However, we visualized the monthly mean night atmospheric conditions from April to August at 850 hPa height (Jiang et al., 2022) over the study area, and the results showed that the monthly mean wind direction blows from north to south, as shown in **Figure 6**. In this study, we simulated more than 680 thousand trajectories and only 4.9% of the trajectories successfully entered the European territory probability density of FAW landing point are shown in **Tables S1**, **S2**. We believed that the invasion of Europe by FAW is still a small probability event, but they might move into Europe with the help of some favorable winds, such as Leveche, Sirocco, and Khamsin.

In summary, it is not a coincidence that FAW invades to Europe, there are suitable environmental conditions every year for FAW to invade southern European countries by wind-driven migration, as well as suitable climatic and vegetation conditions for its survival and reproduction. Southern Europe will be at risk of a cross-sea invasion of FAW when the moth invades and establishes breeding areas along the Mediterranean and Nile coasts of northern Africa. Therefore, there is an urgent need to study the migration characteristics and patterns of FAW, and apply high-precision remote sensing images to provide early warning of the invasion possibility in the key areas of FAW, so that relevant countries can take timely and effective joint control measures to avoid large-scale crop losses caused by FAW in the European region.

## 5 Conclusion

Based on the species distribution model CLIMEX and the numerical trajectory model HYSPLIT, we developed a migration simulation model of FAW to explore the risk, trajectories and periods of invasion of FAW from North Africa to Europe across the Mediterranean Sea from April to August 2016~2022. The annual and seasonal suitable distribution of FAW was first predicted based on climatic suitability, and then the wind-driven migration trajectories of FAW from North Africa to Europe was simulated. Statistical analyses showed that the invasion risk simulations were significantly correlated between years (P<0.005), while the monthly distributions varied widely. Coastal areas are most suitable for the expansion of the FAW, and FAW can invade southern European countries from the Mediterranean coast or the Nile basin and cause damage to local crops such as maize. Spain and Italy have the highest risk of invasion, with 39.08% and 32.20% of effective landing points respectively, followed by Turkey, France, Greece, and Portugal with 8.89%, 6.82%, 5.77%, and 5.08% of effective landings respectively. Our research results will help countries at risk of FAW invasion to make joint control management and develop pest control measures in advance to protect food security.

## Data availability statement

The original contributions presented in the study are included in the article/**Supplementary Material**. Further inquiries can be directed to the corresponding authors.

## Author contributions

JW and YH: data curation, methodology, and writing-original draft. HM and HZ: formal analysis, methodology, and supervision. XZ, XC and YX: investigation and data acquisition. YD and WH: writing-review and editing. LH: conceptualization and funding acquisition. All authors contributed to the article and approved the submitted version.

## Supplementary material

The Supplementary Material for this article can be found onlineat: https://www.frontiersin.org/articles/10.3389/fpls.2023.1141470/full#supplementary-material
