# Supplementary material for: Migration risk of fall armyworm (Spodoptera frugiperda) from North Africa to Southern Europe
Source: Front Plant Sci. 2023 Apr 3;14:1141470. doi: 10.3389/fpls.2023.1141470 (PMC10106561; doi:10.3389/fpls.2023.1141470)
Supplement: Supplementary file 1 [file DataSheet_1.pdf]

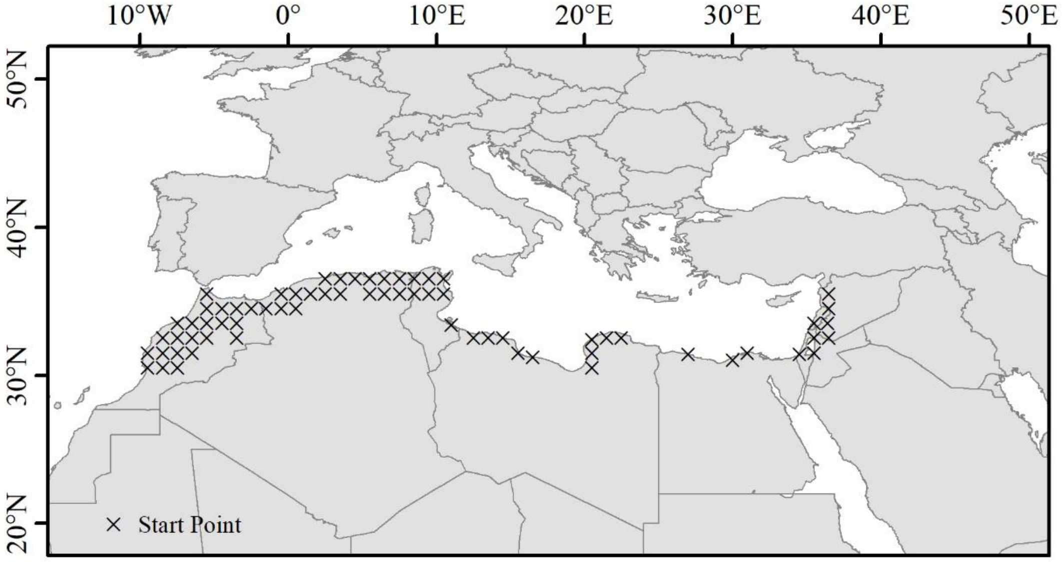

Figure S1. The starting points of the FAW's migration

Table S1. Probability density of FAW landing points by country for 2016-2022

Country	2016	2017	2018	2019	2020	2021	2022
Spain	28.21%	47.40%	36.77%	35.33%	49.84%	27.83%	45.11%
Italy	35.57%	22.86%	29.67%	34.71%	26.87%	46.35%	27.42%
Turkey	14.08%	9.36%	9.57%	8.46%	7.80%	7.58%	7.81%
Portugal	6.10%	7.20%	6.28%	2.61%	3.41%	1.88%	8.26%
France	2.83%	6.78%	3.50%	10.79%	3.14%	8.89%	7.98%
Greece	8.79%	4.62%	11.75%	5.74%	6.14%	5.67%	2.46%
others	4.42%	1.78%	2.46%	2.36%	2.80%	1.80%	0.96%

Table S2. Probability density of FAW landing points by country for April-August

Country	April	May	June	July	August
Spain	11.08%	35.06%	37.76%	52.59%	40.60%
Italy	35.09%	31.44%	35.27%	26.48%	33.90%
Turkey	17.65%	9.76%	6.01%	5.66%	13.84%
Portugal	2.01%	6.12%	6.86%	4.88%	1.36%
France	0.09%	2.78%	9.73%	7.32%	8.48%
Greece	26.11%	11.29%	2.65%	2.22%	1.20%
others	7.97%	3.55%	1.72%	0.85%	0.62%
